# Tai Chi Postures and Joint Health: A Comparative Study of Professional and Amateur Practitioners

**DOI:** 10.1155/abb/5586283

**Published:** 2025-11-20

**Authors:** Cenyi Wang, Xiaohan Wang, Yiwen Liu, Wenbo Wan, Ganfeng Yang, Qinfang Lu

**Affiliations:** ^1^ School of Physical Education and Sports Science, Soochow University, Suzhou, China, scu.edu.tw; ^2^ School of Medicine and Public Service, Suzhou Global Institute, Suzhou, China

**Keywords:** exercise rehabilitation, joint health, sports injury, Tai Chi

## Abstract

This study aimed to compare the knee and ankle joint load characteristics of high and low Tai Chi postures, focusing on three typical Tai Chi movements: Wild Horse Mane (WHM), Repulse Monkey (RM), and Wave Hand in Cloud (WHC). It further explored how different postures affect lower limb loading in practitioners with varying skill levels to provide optimal guidance for Tai Chi practice. A total of 26 male participants were enrolled, divided into the professional group (PG, *n* = 13) and the interest group (IG, *n* = 15). A three‐dimensional (3D) high‐speed motion capture system was employed to record participants’ Tai Chi movements, while a force platform was used concurrently to collect kinematic and dynamic data. For the knee joint, both groups exhibited significantly higher peak moments in the sagittal and coronal planes during the low posture than the high posture across all three movements (*p* < 0.05). During WHM, significant differences in peak ankle moments (sagittal and coronal planes) were noted between the two groups. In RM, the IG showed significantly higher peak ankle moments (sagittal and coronal planes) than the PG (*p* < 0.05). A significant positive correlation was found between posture/skill level and lower limb joint loading, with the knee joint being most affected. Professional practitioners should strengthen the muscles surrounding the knee and ankle joints to enhance joint protection during high‐intensity practice and prevent chronic sports injuries resulting from long‐term joint fatigue. For amateurs, a gradual transition from high to low postures is recommended to adapt to and enhance joint load‐bearing capacity. Additionally, beginners should prioritize ankle flexibility training to improve ankle stability and lower injury risk.

## 1. Introduction

Tai Chi is a traditional Chinese exercise which can promote physical and mental health. It consists of a series of sequential movements involving uninterrupted, slow, and rhythmic weight‐bearing activities. Tai Chi is characterized by slow move, muscle stretching and relaxation, stable rhythm, breath control, and mental concentration during the exercise. At the same time, by varying different postures and speeds, practitioners can adjust Tai Chi to a low or moderate intensity according to their own physical status. Tai Chi is now widely used worldwide as an exercise intervention for rehabilitation and fitness [1].

Long‐term Tai Chi practice benefits practitioners in maintaining good physiological and psychosocial function. Tai Chi stimulates and integrates the musculoskeletal, sensory, and cognitive systems through a series of stable and unstable movements and postures that incorporate balance, coordination, and strength training. Studies have shown that Tai Chi can increase lower limb strength [2], improve flexibility [3], improve balance and postural control [4, 5], and reduce bone density loss [6]. At the same time, Tai Chi has been shown to have a significant positive impact on the treatment and prevention of many age‐related chronic diseases, such as cardiovascular and musculoskeletal disorders [7]. In addition, Tai Chi can be practised anywhere, anytime, at low cost, without tools and in a nonspecific environment.

Yang’s Tai Chi has a smooth rhythm, soft movements and moderate exercise, which is more suitable for people of different ages and physical statuses. Meanwhile, with the widespread of Yang’s Tai Chi movement in the world, many simplified forms of it have been gradually developed to meet the needs of the public. In order to popularize Tai Chi and reduce the difficulty of learning Tai Chi, the Chinese State General Administration of Sport adapted the 24‐form simplified Tai Chi based on characteristics of Yang’s Tai Chi in 1956. And it has now become the most commonly used form of exercise in noncompetitive Tai Chi [8]. Some studies have shown that compared with other types, 24‐form Tai Chi can better improve the motor performance and physical function of elderly chronic patients, such as Parkinson’s disease and Alzheimer’s disease [9]. From the traditional description, Tai Chi gait includes: (1) stepping forward—an anterior movement of one foot in relation to the stance foot, (2) stepping backward—a posterior movement of one foot in relation to the stance foot, (3) stepping sideways—a lateral movement of one foot in relation to the stance foot, (4) up and down stepping—upward lifting of one foot above the knee height of the stance leg, (5) stepping turning—pivotal rotation (medial or lateral) on one stance foot followed by stepping action of the other foot, and (6) stepping fixing, or fixed step—both feet are fixed to the ground with no foot movement [10]. Therefore, the most typical gait patterns in Tai Chi can be summarized, which are forward step, backward step, and side step. During Tai Chi exercise, attention needs to be paid to the control of the center of gravity, and there should not be any big ups and downs. According to the angle of the knee joint, it can be divided into two kinds of poses: a high pose represents a knee joint angle of about 150°, and less than 135° is considered a low pose [11].

However, as Tai Chi continues to evolve in sports rehabilitation practice, some experts begin to hold different views on the pros and cons of Tai Chi practice, particularly with regard to the potential risk of injury due to lower limb joint loading of practitioners. Some studies have shown that excessive lower‐body fatigue occurred during Tai Chi practice may be related to the range of motion (ROM), load intensity, and duration of exercise in the lower limbs. Mechanistically, joint angle is a critical mediator of joint contact force: more flexion angles in weight‐bearing joints concentrate mechanical load onto articular surfaces, directly elevating injury susceptibility [12]. Meanwhile, previous studies indicate that Tai Chi requires the knee joint to remain in a constant flexed position, resulting in elevated mechanical stress and exposing it to risks of pain and injury [13, 14]. This effect is exacerbated in low Tai Chi postures, where increased knee flexion depth and prolonged weight‐bearing further amplify contact force magnitude and cumulative load, aligning with findings on posture‐dependent joint mechanics in martial arts movements [15]. Furthermore, it has been determined that specific Tai Chi movements, namely lunges, pushes, kicks and pseudo‐steps, which are considered to be less conducive to knee health, result in a substantial increase in the load on practitioners’ lower limb joints when compared to slow walking [16]. Consequently, the selection of Tai Chi postures may lead to noticeable discrepancies between the actual effects of the exercise and its intended outcomes [17]. Individuals of varying ages and fitness levels should select movements and postures that are commensurate with their capabilities when practicing Tai Chi.

Therefore, this study will compare the knee and ankle loading characteristics of three classical movements representing forward step, backward step, and side step, respectively, namely Wild Horse’s Mane (WHM), Repulse Monkey (RM), and Wave‐hand in Cloud (WHC) of the 24‐form simplified Tai Chi in high and low poses, and investigate the effects of Tai Chi poses and steps on the knee and ankle loading of Tai Chi practitioners at different skill levels. The findings of this study will provide theoretical references for the development of future exercise programs, with the objective of achieving the optimal exercise effect and reducing the risk of sports injuries.

## 2. Methods

### 2.1. Study Design and Participants

Participants were recruited from Soochow University and divided into two groups: professional group (PG) and interest group (IG). The sample size was calculated by 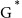
Power 3.1 software. In consideration of the significant effect of gender on lower limb strength, in order to control for potential interference variables, the PG group required male Tai Chi professionals (*n* = 13) with special Tai Chi training background and reached the level of China’s national second‐level athletes (or above); the IG required male Tai Chi amateurs who could skillfully perform 24‐form simplified Tai Chi basic routines (*n* = 15). All subjects had no history of major musculoskeletal or neurological injury in the past 6 months, and the baseline data of subjects in the two groups are shown in Table 1. This study was approved by the Ethics Committee of Soochow University (SUDA20211227H03), and all subjects voluntarily signed informed consent after fully understanding the research purpose and test procedure.

**Table 1:** Baseline information.

Groups	*N*	Age (year)	Body mass (kg)	Height (cm)
PG	13	20.8 ± 1.2	75.4 ± 7.5	175.4 ± 5.4
IG	15	20.6 ± 1.5	73.9 ± 6.9	174.1 ± 6.0

Abbreviations: IG, interest group; PG, professional group.

### 2.2. Experimental Protocol

The three‐dimensional (3D) high‐speed motion capture system used in this study consists of eight infrared cameras (100 Hz, Vicon motion analysis, UK, Figure 1) and is equipped with 35 tracking reflection markers with a diameter of 14 mm for Tai Chi motion capture and acquisition. The position of the marker attachment is made according to the standard scheme for the set of plug‐in gait markers. After the participants entered the experimental site, the same experimenter first explained the whole experimental procedure to them and measured basic data such as height, weight, and length of the left and right lower limbs to reduce experimental errors. Before the formal start of the experiment, each participant was given 5–10 min to warm up with 24‐form simplified Tai Chi exercises to familiarize themselves with the experimental procedure. At the beginning of the formal experiment, the subjects performed three Tai Chi movements in high and low pose: WHM, RM, and WHC. The low pose is defined as the lower limb flexion angle of 135° ± 5°, while the high pose is defined as the maximum knee flexion angle of the supporting leg of 150° ± 5° [11]. The BioVision Multi‐channel Motion Bioelectricity Recording and Analysis System (BioVision, Germany) was used to determine the Tai Chi posture and detect the joint angle in real time, which was placed on the lateral side of the left knee joint. The Tai Chi movements examined in the study included forward step, backward step, and side step. Between each movement, participants were given a 2–4 min rest period to prevent fatigue. The Tai Chi practice was controlled by a metronome at a speed of 30 beats/min. Participants were instructed to step on a force table (100 Hz, 90 cm × 60 cm × 10 cm, model 9287B, Kistler Instrument Corp., Switzerland) with only one foot at a time. It is synchronized with the Vicon infrared high‐speed motion capture system to collect kinematic and dynamic data simultaneously. Data for each Tai Chi movement and posture were recorded and repeated three times for analysis.

Figure 1The Vicon Motion Capture System captured area (green represents the position of the motion capture camera and blue represents the capture coverage space). (a) Camera capture area. (b) Camera placement location.

**Figure:**
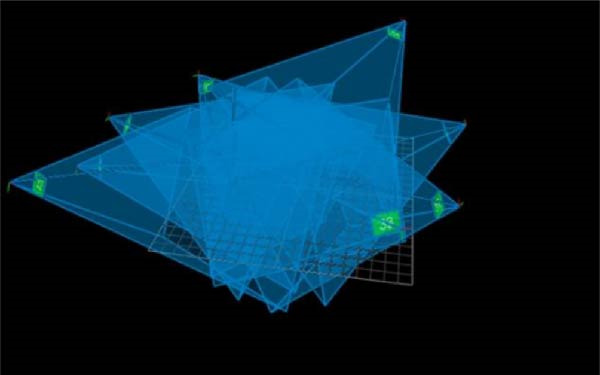
(a)

**Figure:**
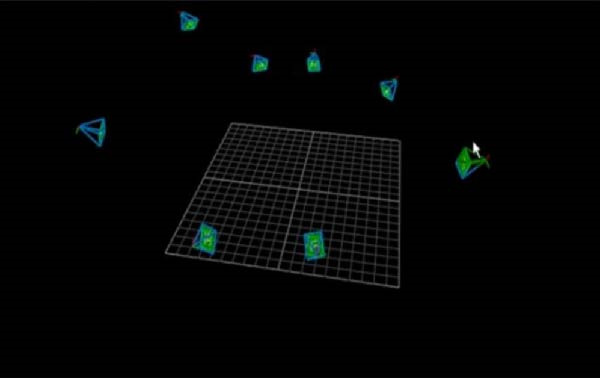
(b)

### 2.3. Data Processing

3D kinematics and dynamics data were calculated using Vicon Nexus (Oxford Metrics, UK) and Visual 3D (C‐Motion, USA) software. Considering that the three Tai Chi movements in this study are symmetrical, the biomechanical characteristics of the left lower limb were selected to represent the whole movement. The kinematic parameters mainly selected include the ROM of the knee and ankle in three planes, and the joint torque and the peak vertical ground reaction force (VGRF) of the left knee and ankle in three directions are selected as the dynamic parameters. A full support phase is defined as the moment when the left foot (left toe) touches the ground and ends when the left toe (left heel) leaves the ground. After three repetitions of each Tai Chi movement and posture, the joint moment and mean VGRF of each participant were averaged and standardized in all directions according to each individual’s body weight (BW).

### 2.4. Statistical Analysis

SPSS 22.0 statistical software (SPSS Inc., IL, USA) was used for descriptive statistics, and the Shapiro–Wilk test was used to test the normal distribution of continuous variables. Two‐factor repeated measurement analysis of variance (ANOVA) was used to analyze the effects of different poses on knee joint and ankle joint load index when normal distribution was followed. Point‐biserial correlation was used to examine the correlation between poses/skill levels and lower limb joint loading indicators separately. According to pose, low pose was expressed as 1 and high pose as 2. According to skill level, high skill was expressed as 1 and low skill as 2. All data were presented as mean ± standard deviation (M ± SD) with a significance level of *p* = 0.05.

## 3. Results

### 3.1. Knee Joint Load Difference

#### 3.1.1. Comparison of Knee Joint Angle Under Different Tai Chi Gait Patterns

As shown in Table 2, there was a significant difference in the coronal plane ROM of the knee joint between two groups of subjects in low pose in WHC gait (*p* < 0.05). A significant difference in the sagittal plane ROM of the knee joint was identified in both groups when comparing different poses within the groups (*p* < 0.05). Additionally, the transverse plane ROM of the knee joint in low pose was found to be significantly greater than that in high pose in WHM gait (*p* < 0.05).

**Table 2:** Comparison of knee joint motion range in different postures (unit: °).

Variables	Pose	PG			IG			Group × task (WHM)		Group × task (RM)		Group × task (WHC)	
		WHM	RM	WHC	WHM	RM	WHC	*F*	*P*	*F*	*P*	*F*	*P*
Flexion and extension ROM	High	41.14 ± 9.03^#^	34.64 ± 3.61^#^	32.12 ± 2.02^#^	49.44 ± 7.05^#^	40.41 ± 8.11^#^	35.63 ± 2.43^#^	2.35	0.14	0.19	0.67	3.28	0.09
	Low	83.96 ± 4.63^#^	65.67 ± 7.68^#^	80.02 ± 4.22^#^	83.96 ± 4.84^#^	73.85 ± 7.43^#^	75.98 ± 10.28^#^						

Abduction and adduction ROM	High	33.74 ± 4.43	25.96 ± 4.04	20.43 ± 4.23	41.40 ± 2.94^#^	38.73 ± 3.02	25.50 ± 5.06^#^	0.02	0.88	2.86	0.11	2.45	0.13
	Low	35.84 ± 5.12	29.42 ± 3.22	25.48 ± 4.44 ^∗^	43.96 ± 1.73^#^	34.54 ± 4.64	36.06 ± 3.37^#^^∗^						

Int. and ext. rotation ROM	High	20.72 ± 2.81^#^	19.93 ± 6.43	18.96 ± 4.72	26.96 ± 7.53^#^	20.35 ± 6.32	19.34 ± 4.53	0.54	0.64	0.47	0.91	1.27	0.26
	Low	25.33 ± 4.38^#^	21.42 ± 2.49	22.48 ± 5.11	33.25 ± 5.74^#^	22.93 ± 3.15	26.33 ± 8.44						

Abbreviations: ext., external; Int., internal.

^∗^indicate significant difference between the groups, *p* < 0.05.

^#^indicate significant difference between the high and the low pose in the same group, *p* < 0.05.

#### 3.1.2. Comparison of Knee Joint Moment Under Different Tai Chi Gait Patterns

**Figure 2:**
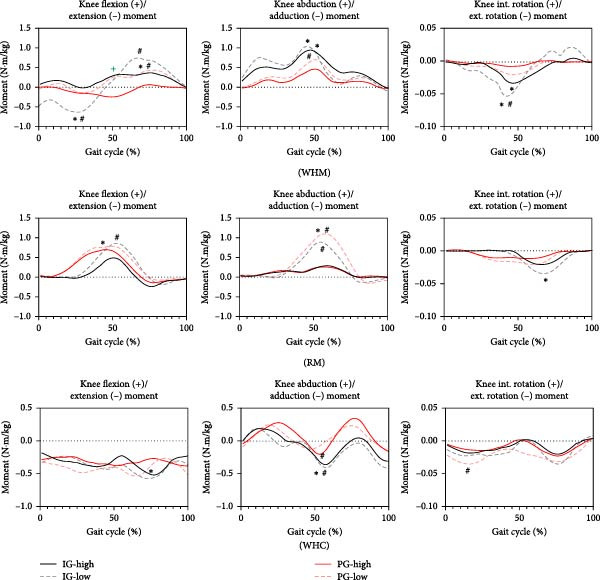
Comparison of knee joint moment in different postures ( ^∗^indicates significant difference between the groups, *p* < 0.05; ^#^indicates significant difference between the high and the low pose in the same group, *p* < 0.05; ^+^indicates interaction effects between poses and groups, *p* < 0.05).

As shown in Figure 2, the within‐group analyses showed that there was a significant difference in peak knee flexion–extension moment in different poses between two groups, with low pose significantly higher than high pose (*p* < 0.05). Interaction analysis showed that there was an interaction effect between group and posture for WHM gait (*p* < 0.05). Within‐group analysis demonstrated that the peak knee abduction moment in low pose was significantly higher than that in high pose in the PG (*p* < 0.05). The peak knee rotation moment was significantly higher in the IG than in the PG (*p* < 0.05).

In RM gait, the within‐group analyses demonstrated that the peak knee flexion–extension moment in low pose was significantly higher than that in high pose in the IG (*p* < 0.05). In addition, there was a significant difference in peak knee abduction moment between two groups in low pose, which was significantly higher in the PG than in the IG (*p* < 0.05).

In WHC gait, there was a significant difference in peak knee flexion–extension moment between the PG and the IG in high pose, with the IG significantly higher than the PG (*p* < 0.05), whereas the within‐group analysis did not show any significant difference (*p* < 0.05). In addition, there was a significant difference in peak knee joint adduction moment in low pose between two groups (*p* < 0.05). Within‐group analysis demonstrated that peak knee joint adduction moment in low pose was significantly higher than that in high pose in both groups (*p* < 0.05).

### 3.2. Ankle Joint Load Difference

#### 3.2.1. Comparison of Ankle Joint Angle Under Different Tai Chi Gait Patterns

As shown in Table 3, there was a significant difference in the coronal plane ROM of the ankle joint between two groups in high pose in WHM gait (*p* < 0.05), and of the transverse plane ROM in both high and low pose (*p* < 0.05). Comparison of different postures within the group showed that there was a significant difference in transverse plane ROM of ankle in both of groups in different postures in WHC gait (*p* < 0.05). The IG had significantly greater ankle transverse plane ROM in low pose than in high pose in WHM gait (*p* < 0.05). The results of the interaction effect showed that there was an interaction between group and posture in knee coronal plane ROM in WHM gait (*p* < 0.05).

**Table 3:** Comparison of ankle joint motion range in different postures (unit: °).

Variables	Pose	PG			IG			Group × task (WHM)		Group × task (RM)		Group × Task (WHC)	
		WHM	RM	WHC	WHM	RM	WHC	*F*	*P*	*F*	*P*	*F*	*P*
Dorsi. and plantar. ROM	High	67.26 ± 4.58	83.48 ± 4.42	79.68 ± 5.38	68.93 ± 6.37	67.26 ± 4.58	55.60 ± 2.38	0.19	0.67	0.95	0.35	0.68	0.54
	Low	79.80 ± 2.44	90.73 ± 7.19	90.14 ± 10.38	79.63 ± 7.50	69.30 ± 5.24	55.74 ± 10.96						

Abduction and adduction ROM	High	5.11 ± 1.12 ^∗^^**+**^	10.59 ± 3.84	3.53 ± 1.21	7.78 ± 1.49 ^∗^^**+**^	5.97 ± 0.98	4.29 ± 1.41	5.87	0.03	0.34	0.72	0.92	0.39
	Low	6.84 ± 0.50^+^	8.72 ± 2.07	4.86 ± 1.91	7.34 ± 1.03^**+**^	7.27 ± 2.35	6.86 ± 2.15						

Int. and ext. rotation ROM	High	53.01 ± 4.38 ^∗^	55.28 ± 7.32	37.97 ± 8.67^#^^∗^	32.54 ± 6.91 ^∗^^#^	43.23 ± 3.58	29.4 ± 2.48^#^^∗^	0.68	0.53	0.54	0.59	0.64	0.56
	Low	55.32 ± 7.38 ^∗^	57.20 ± 9.29 ^∗^	43.52 ± 7.40^#^^∗^	40.36 ± 9.54 ^∗^^#^	38.48 ± 6.12 ^∗^	34.55 ± 3.74^#^^∗^						

Abbreviations: Dorsi., dorsiflexion; ext., external; Int., internal; plantar., plantarflexion.

^∗^indicate significant difference between the groups, *p* < 0.05.

^#^indicate significant difference between the high and the low pose in the same group, *p* < 0.05.

^**+**^indicate interaction effects between poses and groups, *p* < 0.05.

#### 3.2.2. Comparison of Ankle Joint Moment Under Different Tai Chi Gait Patterns

**Figure 3:**
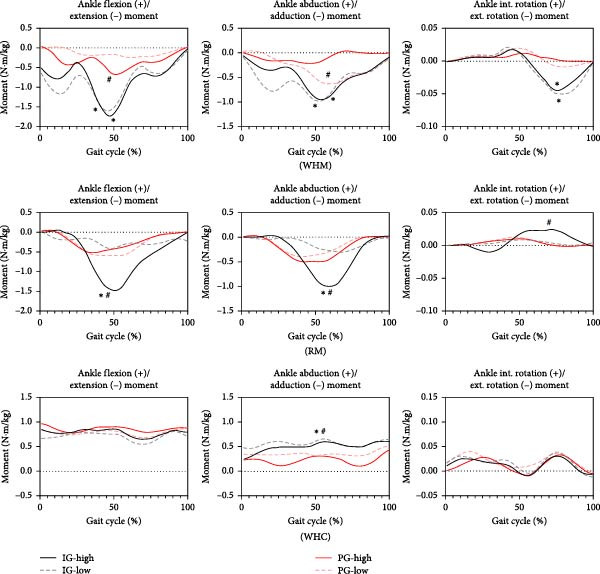
Comparison of ankle joint moment in different postures ( ^∗^indicates significant difference between the groups, *p* < 0.05; ^#^indicates significant difference between the high and the low pose in the same group, *p* < 0.05).

As shown in Figure 3, there was a significant difference in peak ankle flexion–extension moment of two groups in WHM gait, with the IG significantly higher than the PG (*p* < 0.05). Within‐group analysis showed that peak ankle flexion–extension moment was significantly higher in low pose than in high pose in the PG (*p* < 0.05). In addition, there was a significant difference in peak ankle abduction moment between two groups (*p* < 0.05).

In RM gait, the within‐group analyses showed that the peak ankle flexion–extension moment was significantly higher in high pose than in low pose within the IG (*p* < 0.05). In addition, there was a significant difference in peak ankle abduction moment between two groups in low pose, with the IG higher than the PG (*p* < 0.05). Within‐group analyses demonstrated that there was a significant difference in peak ankle rotation moment between high and low pose in the IG (*p* < 0.05). In WHC gait, peak ankle abduction moment was significantly higher in the IG than in the PG in different postures (*p* < 0.05).

### 3.3. VGRF Difference

**Figure 4:**
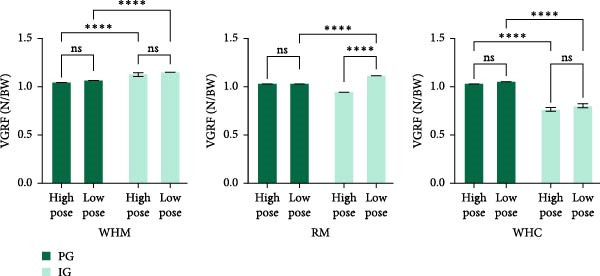
Comparison of VGRF in different postures.  ^∗∗∗∗^ *p* < 0.001, ns means *p* > 0.05.

As shown in Figure 4, in WHM gait, VGRF was significantly higher in both high and low poses in the IG than in the PG (*p* < 0.05), whereas no significant difference in VGRF was observed in different postures within the IG (*p* < 0.05). In RM gait, VGRF was significantly higher in the IG than in the PG in low pose (*p* < 0.05), and it was significantly greater in low pose than in high pose in the IG (*p* < 0.05). In WHC gait, there was a significant difference between the VGRF of both groups in high and low pose (*p* < 0.05), while no significant difference was seen between VGRFs in different postures within two groups (*p* < 0.05).

### 3.4. Correlation Between Lower Limb Load and Posture, Skill Level

As shown in Figure 5, in WHM gait, the Tai Chi pose was significantly correlated with knee sagittal plane ROM, horizontal plane ROM, knee peak joint moment, and ankle coronal plane ROM with peak joint moment (*p* < 0.05). In RM gait, the Tai Chi pose was significantly correlated with knee sagittal plane ROM, knee sagittal and coronal plane peak moments, and ankle coronal plane peak moments (*p* < 0.05). In WHC gait, the Tai Chi pose was significantly correlated with knee sagittal plane ROM, knee coronal plane ROM, knee coronal plane peak moment, and ankle horizontal plane ROM (*p* < 0.05).

Figure 5Point biserial correlation of lower limb load with pose and skill level (a) correlation analysis of knee joint load and VGRF. (b). correlation analysis of ankle joint load. *X*: sagittal plane, *Y*: coronal plane, *Z*: transverse plane).

**Figure:**
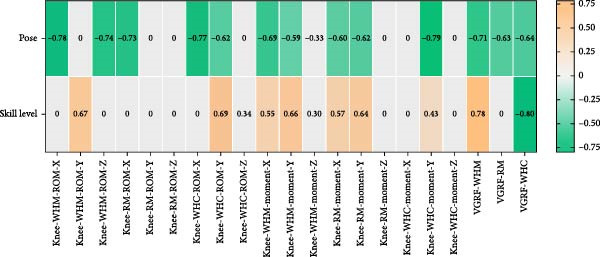
(a)

**Figure:**
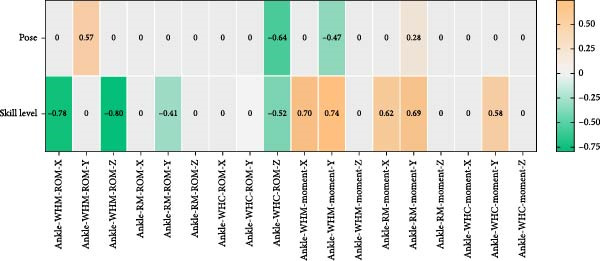
(b)

In WHM gait, skill level was significantly correlated with knee coronal plane ROM, knee peak joint moments, ankle sagittal plane ROM, horizontal plane ROM, peak sagittal plane moments, and peak coronal plane moments (*p* < 0.05). In RM gait, skill level was significantly correlated with peak knee sagittal plane moment, peak coronal plane moment, ankle sagittal plane ROM, peak ankle sagittal plane moment, and peak coronal plane moment (*p* < 0.05). In WHC gait, skill level was significantly correlated with knee coronal ROM, horizontal plane ROM, peak coronal plane moment, ankle horizontal plane ROM, and peak coronal plane moment (*p* < 0.05).

## 4. Discussion

### 4.1. The Influence of Posture on the Knee Joint

In our in‐depth study of the effects of Tai Chi postures on the lower limb joints of practitioners, we focused on the biomechanical properties of the knee and ankle joints of practitioners with different Tai Chi skill levels under different Tai Chi gaits and postures, revealing the complexity of joint activities during Tai Chi practice and their potential impact on the health of the knee joint.

The results of this study demonstrated that the low pose significantly increased the flexion and extension joint ROM of the knee, which was particularly evident in WHM and RM gaits. The low pose requires the practitioner to squat deeper and have a greater knee flexion angle, which increases the contact stresses and loads on the articular surfaces. Previous studies have shown that the knee joint has the smallest articular surface contact area and the greatest force per unit area at flexion angles close to 90°, which may lead to accelerated wear and tear of the articular cartilage [18]. Although this increased ROM can help to build lower limb muscular strength and improve body stability, prolonged repetitive high‐load exercises, even for professional Tai Chi practitioners at a high level of skill, also increase the risk of cumulative knee injury, such as articular cartilage wear and ligament strains [13]. Meanwhile, in the low pose of WHM gait, there was a significant difference in knee coronal plane ROM in both groups compared to that in the high pose, which suggests that WHM gait in the low pose required a greater knee adduction or abduction angle to accommodate the transfer of the center of gravity and to maintain balance. Notably, the IG also had significantly greater knee transverse plane ROM in the low pose of the WHM gait compared to the high pose, suggesting that nonprofessional Tai Chi practitioners in this gait are more likely to experience knee hypermobility in the low pose and that the body requires more lateral movement to control the movement and posture. In contrast, the Tai Chi PG may have more precise knee control in low pose due to long‐term professional physical training, which reduces unnecessary overactivity. The WHM gait is one of the most common movements in Tai Chi, and the elderly and frail populations need to pay attention to the control of the peripheral knee muscles to reduce the risk of sports injuries when improving the difficulty of Tai Chi by lowering the pose [19].

Joint moment and VGRF are used as important parameters for assessing lower limb loading. Their changes directly reflect the muscle force output and the magnitude of the impact force endured by the joints during practice. In the present study, we observed a significant increase in peak knee joint moments during the execution of WHM, RM and WHC gaits in Tai Chi practitioners in low pose, which is consistent with previous studies [20]. This change in knee joint moment reflects an increase in the muscular forces required to maintain stability and complete Tai Chi movements, especially in the major lower limb muscle groups, such as quadriceps and hamstrings, which are required to generate a larger centrifugal contraction force to control the flexion and extension of the knee joints in low pose [21]. In addition, there was an interaction effect between group and pose for peak knee moment in WHM gait, indicating that there were differences in the performance of knee flexion and extension forces between two groups of practitioners performing the same pose, which has been linked to muscle strength, technical proficiency, and practice experience. Incorrect WHM gait results in greater knee extensor moments, as well as knee abduction and external rotation moments. The results of the correlation between pose, skill level, and knee loading in this study also reflect the possible large differences in the synergistic patterns of action of the periprosthetic knee muscle groups between the two poses for practitioners of different skill levels.

In WHM and RM gaits, the VGRF of the PG was significantly lower than that of the IG, and this difference reflects that professional practitioners are able to better control the transfer of the body’s center of gravity, reduce the ground reaction force, and improve the efficiency of the movement through long‐term training, thus reducing the burden on the knee joint and other lower limb joints. However, the results of WHC gait in this study demonstrated that the VGRF of the PG was higher than that of the IG. Considering that this result relates to the characteristics of WHC gait, which emphasizes rotation of the body and lateral and continuous transfer of the center of gravity, maintaining the accuracy of movements in low pose requires a higher level of balance and coordination in addition to stable lower limb power output [22]. The general practitioners usually tend to pass through the postural movements when they complete the low pose of the movement. The average practitioner can easily compensate for low pose by contorting the posture and shortening the standing time on one foot. Among the three typical movements, the results of this study demonstrated that the VGRF of IG subjects in low pose was higher than that in high pose only in RM gait, and no significant difference was observed in the remaining movements, which is different from the expectation that low pose of Tai Chi is usually considered to significantly increase the ground reaction force in completing the Tai Chi movement [20]. However, the findings in this study may illustrate that professional Tai Chi practitioners in different gaits are able to effectively utilize lower limb muscle power to adjust body pose and power distribution, demonstrating the ability to flexibly adjust power output.

### 4.2. The Influence of Posture on the Ankle Joint

In this study, it was found that there was a significant difference in the ankle coronal plane ROM (internal and external rotation) between two groups in the high pose of WHM gait, whereas in the transverse plane ROM (pronation and supination), both the high and low poses of both WHM and WHC gaits showed significant between‐group differences. This suggests that high poses of Tai Chi places more emphasis on ankle flexibility in the coronal plane, such as adjusting to maintain body balance and movement fluidity through internal and external rotation of the foot, whereas low poses could increase the need for movement in the horizontal plane, such as greater foot translation to support the transfer of BW. The PG demonstrated greater flexibility and control in these variations, which further demonstrates the positive effects of increased motor skill levels on ankle function [23].

In this study, the differences in peak ankle flexion, extension and abduction moment not only reflected the specific requirements of Tai Chi movements for joint strength but also reflected the significant differences in power output between professional and nonprofessional practitioners. In WHM gait, the significant differences in peak ankle flexion, extension, and abduction moment between the PG and IG reflect the superiority of professional practitioners in ankle strength output. The difference in the flexion and extension moment could be attributed to the excessive single‐leg support movements observed in Tai Chi, such as leg cocking and drop step, which require optimal power performance of the ankle joint to support the rapid change of the body’s center of gravity in the front and rear. Conversely, the observed variations in abduction moment appear to be associated with the maintenance of lateral stability, particularly during execution of movements such as WHC that necessitate lateral body shifts. This finding is consistent with the previous results concerning lateral joint angles. Although peak ankle rotational moment did not show significant differences between poses in this study, this does not mean that rotational moment is not important in Tai Chi. On the contrary, rotational movements in Tai Chi rely more on the drive of the waist and hips and the overall coordination of the lower limbs than on the rotation of a single ankle joint [24]. This coordinating mechanism reduces the individual loading of the ankle joint and improves the stability and efficiency of the overall movement [25]. The relative stability of Tai Chi practitioners in rotational moment can thus be a reflection of their overall coordinating ability.

The synergistic work of the knee and ankle joint in Tai Chi movement is the key to achieving smoothness and stability of movement [26]. Through the rapid response and precise regulation of the nervous system, the PG is able to adjust the joint ROM and force output in real time compared with the IG to adapt to the different demands of the movement. At the same time, the feedback mechanism between the joints helps the practitioner to sense and correct deviations and errors in the movement. The stability and flexibility of the knee joint, as the main load‐bearing joint of the lower limb, directly affects the load sharing and performance of the ankle joint [27]. Similarly, the flexibility and power output of the ankle joint in turn affect the loading and stability of the knee joint [28]. In the low pose of Tai Chi, when the knee joint is subjected to excessive loads, the neural system quickly transmits signals to the ankle joint and other peripheral joints and muscles to make load‐sharing and stability adjustments. Full dorsiflexion of the ankle reduces the load‐bearing pressure on the knee, while adduction and rotation of the ankle help to maintain balance and stability in the lateral movement. Recent studies have concluded that Tai Chi can improve the muscle groups and neural control sensitivity of the ankle joint of the lower limb, especially the deeper part of the knee [29]. However, it is worth noting that in this study, the peak ankle flexion and extension moment and peak abduction moment of the IG were significantly higher in high pose than in low pose in RM gait. Typically the ankle moments in low pose are greater due to the deeper body squat, which requires more stability and strength in the ankle joint. Consider that the IG may have relied too much on thigh and hip strength to maintain balance in the low pose and neglected the ankle joint, thus reducing the ankle moment output (negative results explained). Furthermore, the magnitude of the ankle moment is closely related to the strength of the calf muscles [30]. In the event of novice individuals possessing deficient calf muscles and poor ankle mobility, they may encounter difficulties in achieving a full ROM in a low posture when executing maneuvers necessitating a higher level of balance, such as a “backward step.” This results in a restriction of their ankle mobility. The lower ankle ROM in the RM gait of IG in this study corresponds to this.

There are a number of limitations to this study. First, the sample size was relatively small and focused mainly on specific age and gender groups, which may limit the generalisability and representativeness of the findings. Future studies should consider expanding the sample size to cover practitioners of different ages, genders, fitness levels, and training backgrounds in order to more comprehensively assess the effects of Tai Chi on lower limb joints. In addition, this study mainly used biomechanical indexes to assess the function and performance of the knee and ankle joint but failed to fully consider the combined effects of physiological and psychosocial factors. Future studies could incorporate multidisciplinary perspectives, such as physiology and sociology to explore in depth the comprehensive effects of Tai Chi on the lower limb joints of practitioners.

## 5. Conclusion

The pose of all three typical Tai Chi gaits could significantly affect the knee and ankle joint loads of the practitioners. Despite demonstrating superior control and adaptability with respect to knee and ankle flexibility, stability, and power output in comparison to nonprofessionals in a lower pose, the observed discrepancy may be attributable to the enhancement of neuromuscular control that is a consequence of prolonged professional training. It is vital to recognize that long‐term practice with high loads may incur a heightened risk of cumulative injuries.

Professional practitioners should lay emphasis on strengthening the muscle groups around knee and ankle joints in order to further improve the joint protection ability during high‐intensity practice and avoid chronic injuries to knee and ankle joints caused by long‐term fatigue. For Tai Chi enthusiasts, it is recommended to gradually transition from high pose to low pose in order to gradually adapt to and improve the loading capacity of the joints. At the same time, beginners should pay special attention to ankle flexibility training to enhance the support and stability of the ankle joint and reduce the risk of injury. This research provides a theoretical basis for formulating scientific and personalized Tai Chi training programs for Tai Chi practitioners. Future studies should focus on longitudinal designs to track the long‐term adaptations and potential risks associated with sustained low‐posture training.

## Consent

Written informed consent was obtained from the participant for publication of this study.

## Disclosure

The funder had and will not have a role in any of the aspects in the study design, data collection analysis, publication, or development of the manuscript.

## Conflicts of Interest

The authors declare no conflicts of interest.

## Author Contributions

**Cenyi Wang**, **Xiaohan Wang**, and **Yiwen Liu**: writing – original draft, writing – review and editing. **Ganfeng Yang** and **Qinfang Lu**: supervision, writing – original draft, writing – review and editing. **Wenbo Wan** and **Qinfang Lu**: conceptualization, supervision, writing – original draft, writing – review and editing.

## Funding

This research was supported by the Postdoctoral Fellowship Program of CPSF (Grant GZC20231899) and the Major Sports Research Project of Jiangsu Sports Bureau (Grant ST242106).

## Data Availability

The data that support the findings of this study are available from the corresponding author upon reasonable request.
